# Normal values of exhaled carbon monoxide in healthy subjects: comparison between two methods of assessment

**DOI:** 10.1186/1471-2466-14-204

**Published:** 2014-12-16

**Authors:** Umberto Moscato, Andrea Poscia, Riccardo Gargaruti, Giovanni Capelli, Franco Cavaliere

**Affiliations:** Institute of Public Health, Hygiene Division, Catholic University “Sacro Cuore”, Largo Francesco Vito, 1, 00168 Rome, Italy; Institute of Anaesthesia and Intensive Care, Catholic University “Sacro Cuore”, Rome, Italy; Institute of Hygiene, University of Cassino, Cassino, Italy

**Keywords:** Carbon monoxide, Exhaled carbon monoxide, Photo-acoustic spectrometer, Electrochemical analyser

## Abstract

**Background:**

In a previous study, exhaled carbon monoxide (eCO) has been assessed in healthy non-smokers with a photo acoustic spectrometer Brüel&Kjær 1312. Unexpectedly, values were higher than those reported in literature, which were mostly obtained with electrochemical analysers. This study was aimed to compare eCO values obtained with Brüel&Kjær 1312 and PiCO + Smokerlyzer, a largely utilized electrochemical analyser.

**Methods:**

Thirty-four healthy subjects, 15 non-smokers and 19 smokers, underwent eCO assessment with Brüel&Kjær 1312 and PiCO + Smokerlyzer during a prolonged expiration (15 seconds). Brüel&Kjær 1312 assessed CO concentration 7 and 12 seconds after the beginning of expiration and displayed the mean value. PiCO + Smokerlyzer was utilized according to the manufacturer’s recommendations. In vitro, the two devices were tested with standard concentrations of CO in nitrogen (5, 9.9, 20, and 50 ppm), and the time needed by PiCO + Smokerlyzer readings to stabilize was assessed at different gas flows.

**Results:**

Both Brüel&Kjær 1312 and PiCO + Smokerlyzer presented very good internal consistency. The values provided were strictly correlated, but at low test concentrations, the Brüel&Kjær 1312 readings were greater than the PiCO + Smokerlyzer, and vice versa. PiCO + Smokerlyzer overestimated the CO standard concentrations at 5 and 9.9 ppm by 20%, while Brüel&Kjær 1312 measures were correct. PiCO + Smokerlyzer readings stabilized in 12 seconds during in vitro tests and in 15 seconds during in vivo measurements, suggesting that the values displayed corresponded to the initial phase of expiration.

**Conclusions:**

Differences between Brüel&Kjær 1312 and PiCO + Smokerlyzer may be explained because Brüel&Kjær 1312 measured CO levels in the middle and at the end of expiration while PiCO + Smokerlyzer assessed them in the initial part of expiration.

## Background

Carbon monoxide (CO) is the product of the heme conversion to biliverdin by microsomal heme oxygenase; a further amount (about 15%) results from the degradation of myoglobin, guanylyl cyclase, and cytochromes [1]. In the human body, CO is not simply a waste product of heme metabolism, but also a neurotransmitter and has important anti-inflammatory, anti-proliferative, anti-apoptotic, and antioxidant properties [2]. The amount of CO stored in the body is affected by endogenous and exogenous factors [3]. In the presence of hemolysis, the rate of heme conversion to biliverdin increases [4]. In local and systemic inflammatory states, an inducible isoform of heme oxygenase (OH-1) is synthetized, which increases the rate of heme metabolism and, consequently, CO production. In smokers, CO produced during the combustion of tobacco is partly absorbed through the inhaled air [5]. Since CO is mainly removed from the body through the lungs, its concentration in the exhaled air (eCO) increases whenever one of these conditions occurs.

Increased eCO values have been reported in systemic diseases, such as severe sepsis [6, 7], cystic fibrosis [8], cirrhosis [9], and after liver transplantation [10]. Increases have also been observed in some respiratory diseases, such as asthma [11–13], inflammatory pulmonary diseases [14], upper and lower respiratory tract infections [15, 16], bronchiectasis [17], seasonal allergic rhinitis [18], and in lung transplantation [19], probably as the result of local inflammation [20, 21]. The potential usefulness of eCO assessment in these conditions is increased by the ease of measurement, which can be carried out by portable and reasonably priced devices that utilize electrochemical sensors and offer good levels of sensitivity (usual determination limit: 1 ppm).

Recently, a photo acoustic spectrometer was utilized to investigate the influence of hypoventilation and hyperventilation on eCO levels in a group of healthy volunteers [22]. Such device was chosen because it is linear over a wide dynamic range and provides higher levels of sensitivity (detection limit up to 0.02 ppm) than traditional CO analyzers based on electrochemical sensors. The results of the study showed that the values obtained with the photo acoustic spectrometer, although characterized by very good internal consistency, were above the range considered normal for the CO analyzers based on electrochemical sensors.

The aim of this study was to compare eCO measures provided by a photoacustic spectrometer and an electrochemical analyzer in vivo and in vitro in order to assess the comparability of use of the two detection systems.

## Methods

### Subjects

After obtaining the approval of the local Ethics Committee of the Catholic University “Sacro Cuore” and the informed consent, 34 healthy volunteers (29.5 ± 6.5 years; BMI 22.5 ± 3.7 Kg/m^2^) were recruited. Eight males and 11 females were current smokers (Median 10 cigarettes/day; Range 2–30 cigarettes/day), while 7 males and 8 females were non-smokers. Non-smokers were defined as subjects without a story of active or passive smoking in the previous 4 months. None of the subjects had undergone previous eCO measurements with the two devices tested in the study. Exclusion criteria were a medical history of acute or chronic respiratory inflammatory diseases, and the ingestion of anti-inflammatory drugs during the previous 72 hours.

### Devices

The characteristics of the two devices were as follows:

- Brüel&Kjær 1312 (B&K) (Brüel & Kjær, AirTech Instruments, Ballerup, Denmark) is a field gas monitor based on the photo-acoustic effect [23]. The device performs a side stream analysis in a measurement chamber, where the molecules of CO absorb energy from monochromatic infrared light. Energy absorption increases the kinetic energy of the molecules and causes the generation of sound waves, which are detected by a stable transducer (microphone). The device is characterized by a very low detection limit (less than 0.02 ppm) and provides automatic compensation for water vapour and other gas interference, and for temperature. The gas mixture to be analysed is aspirated into the measurement chamber at a constant rate through a Teflon/Viton tube one meter long; sampling begins after the washout of the dead space. For each measurement, the operator sets the duration and volume of sampling and the frequency of analysis. In this study, sampling lasted 10 seconds and two measures were performed, at 5 and 10 seconds; only the mean value was displayed by the device. According to the manufacturer’s recommendations, sampling volume was determined with the following algorithm:

V_1302_ = internal volume of the device circuits = 50 mL

V_PMC_ = Photoacustic Measurement Chamber volume ≈ 3 mL

V_tube_ = volume of the sampling tube = 3.14 × 0.15^2^ × 100 = 7.07 mL

b) Bedfont EC50 PiCO + Smokerlyzer (PiCO+) (Bedfont Scientific, Kent, UK) is a small, portable device primarily aimed to distinguish smokers from non-smokers and to classify smokers on the basis of their smoking habits. It has been also utilized in many studies that investigated eCO levels in respiratory diseases [8, 9, 11–18]. This instrument utilizes an electrochemical sensor to analyse exhaled air for eCO concentration and has a detection limit of 1 ppm. Displayed values increase until a stable reading is reached. According to the manufacturer’s recommendations, in this study measurements were performed during a sustained exhalation after a deep breath to total lung capacity, followed by a 15 s breath-hold.

### Protocol

**Figure 1:**
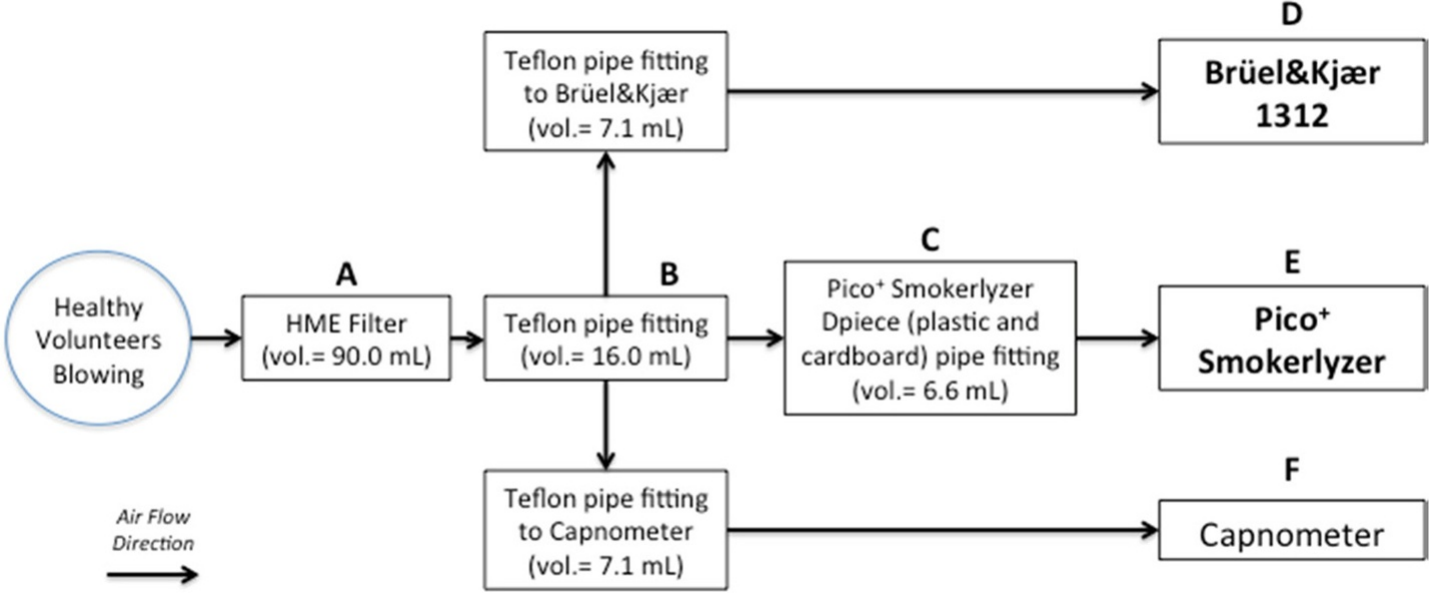
**Diagram of the experimental circuit and volumes of the components.** The subjects enrolled in the study were asked to breathe through a circuit consisting of **(A)** an HME bacterial/ viral filter (DAR Barrierbac S, Mallinkrodt DAR, Italy); **(B)** Teflon connection tube, with three output ports, connected to: **(C)** cardboard and plastic tube (D-piece) through which the exhaled gas was sent to **(E)** PiCO + Smokerlyzer; **(F)** capnometer CosmoPlus mod. 8100 (Novametrix Medical Systems Inc. Connecticut, USA); **(D)** photoacoustic spectrophotometer Brüel&Kjær 1312. The direction of the arrows points out the direction of air flow.

1. In vivo comparison between B&K and PiCO+After recording demographic and anthropometric data and medical history, all subjects underwent a brief training on the procedure. They were asked to perform a deep breath to total lung capacity, a 15 s breath-hold, and a 15 s sustained exhalation through a mouthpiece. The mouthpiece was connected to a circuit that in the order consisted of a HME bacterial/viral filter (DAR Barrierbac S, Mallinkrodt DAR, Italy), a Teflon connection tube, with three output ports, connected to: a cardboard and plastic tube through which the exhaled gas was sent to the PiCO+; a capnometer CosmoPlus mod. 8100 (Novametrix Medical Systems Inc. Connecticut, USA); a photoacoustic spectrophotometer B&K (Figure 1). Gas sampling for B&K took ten seconds and started 2 seconds after the beginning of exhalation in order to discard the anatomical dead space (such delay was chosen on the basis of preliminary tests). Accordingly, CO concentration was assessed seven and twelve seconds following the beginning of expiration. During each measurement, the operator checked that the plateau phase of the capnogram was already started when gas sampling began. In each subject, three valid measures by B & K and PiCO+ were obtained.

Prior to starting the study, both B&K and PiCO+ were calibrated according to the manufacturer’s recommendations.

2) In vitro tests
  A first series of measurements were performed by connecting the previously described circuit to cylinders that contained mixtures of CO and nitrogen (Sapio Srl, Italy). The CO concentrations tested were 5, 9.9, 20, and 50 ppm. The pressure of gas outlet was set at 0.12 bar. The means of 4 measurements were calculated.
  A second set of measurements was performed in order to relate PiCO+ readings and latency to gas flows. Measurements were performed with the cylinder that contained 9.9 ppm of CO by varying outlet pressure from 0.01 to 0.12 bar. Flows were assumed to be proportional to the pressures applied to the circuit because a linear flow could be reasonably hypothesized. The eCO values provided by PiCO+ and the time elapsed from the beginning of expiration to the appearance of stable readings were recorded.

### Statistics

Data are presented as means (standard deviations) or medians (ranges) as appropriate. Wilcoxon signed-ranks test was performed to test the difference between the two instruments both in vivo and in vitro. Intraclass correlation coefficients (ICCs) were utilized to compare the measures obtained by the two devices tested. Bland-Altman plots were also employed to describe the limits of agreement to data with repeated measurements (for equal numbers of replicates by each method on each subject) [24].

## Results

All subjects successfully performed three sets of measurements. The mean number of attempts required to obtain three approved measurements was 4.2 (0.9). No adverse event was observed. Environmental CO concentration measured with B&K was 1 (0.2) ppm.

**Figure 2:**
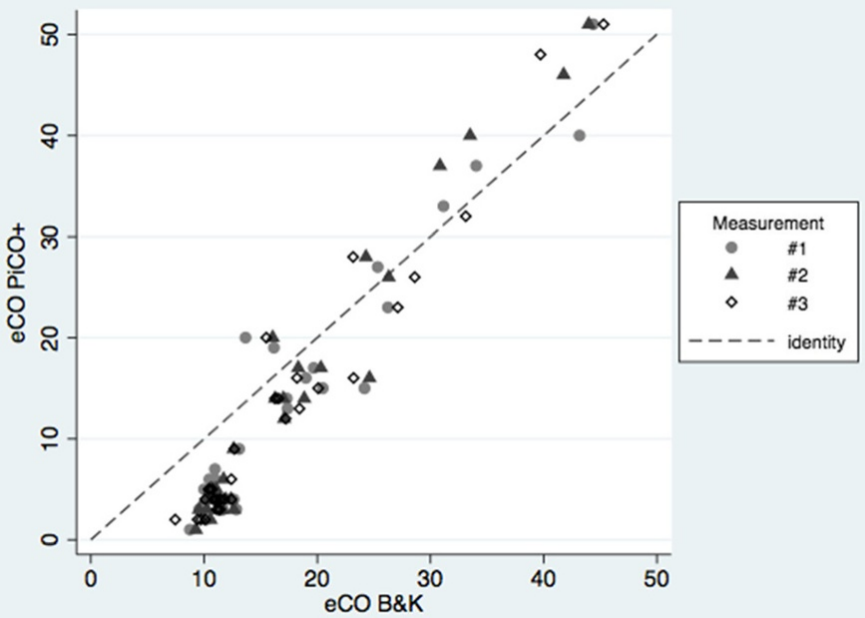
**Correlation between eCO values (ppm), obtained with Brüel&Kjær 1312 and PiCO + Smokerlyzer, in the subjects enrolled in the study.** The measures taken into account were 102 (3 measurements for each of 34 subjects). The Identity line (Y = X; grey dashed line) is reported.

**Figure 3:**
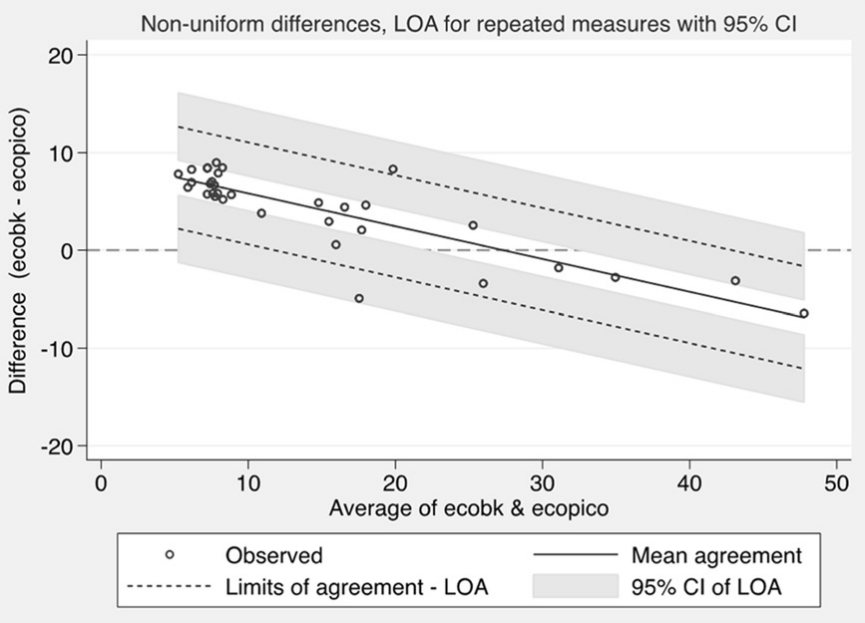
**Agreement between Brüel&Kjær 1312 (eCOBK) and PiCO + Smokerlyzer (eCOPiCO) (Bland Altman plot).** The measures taken into account were 102 (3 measurements for each of 34 subjects). The regression line for the mean agreement (Y = −0.3336 X −9.192) is reported. The coefficient of determination R^2^ was ≈ 0.7214 (p = 0.000). The standard deviation of the residuals is 2.2716; there is no significant relationship between the standard deviation of the differences and the average of the two methods (p = 0.165). To consider the repeated measures approach and the non linear relationship between difference and mean, the Limits of Agreement (LOA) was evaluated modelling the variability in the Standard Deviation of the mean difference directly as a function of the level of the measurement.

1. In vivo comparison between B&K and PiCO+
  Table 1 presents eCO values measured with B&K and PiCO+. Data are stratified by gender and smoking habit. The eCO values obtained by B&K were significantly higher than those obtained by PiCO+ (12.2 (7.4 – 45.3) Vs. 5.0 (1 – 51) ppm) (p <0.01). Stratified data did not point out differences related to gender, but values were higher in smokers than in non-smokers (p <0.001). Both the devices presented an excellent internal consistency. Intraclass correlation coefficients were 0.995 for B&K and 0.985 for PiCO+ (estimated reliability 0.998 and 0.995). PiCO+ displayed the initial value after 3 (1) sec and the final value after 15 (2) sec in non-smokers and after 3 (1) sec and 20 (3) sec in smokers.
  The values obtained by the two instruments were strongly correlated (Figure 2). The Bland-Altman plot (Figure 3) showed that the relationship between difference and mean is not linear and that the gap was dependent on eCO levels. In particular, B&K displayed higher values than PiCO+ at low eCO levels and lower values at high eCO levels; measures were practically equivalent in the interval between 20 and 40 pm. The mean difference and the limits of agreement for repeated measures adjusted for non uniform differences and their 95% CI are shown in Figure 3.

**Table 1:** **eCO values (ppm) provided by B&K and PiCO+ in the healthy volunteers enrolled in this study**

	Smokers			Non-smokers		
	Males	Females	Total	Males	Females	Total
	N = 8	N = 11	N = 19	N = 7	N = 8	N = 15
**B&K**	21.8	16.3	18.3	11.2	10.4	10.9
	(31.71)	(28.35)	(28.35)	(2.15)	(4.16)	(5.17)
**PiCO+**	23.8	15.9	19.2	4.3	3.2	3.7
	(13.27)	(12.94)	(13.54)	(4.5)	(3.4)	(3.4)

Values are stratified by gender and smoking habits and reported as medians (ranges: max-min values).

**Figure 4:**
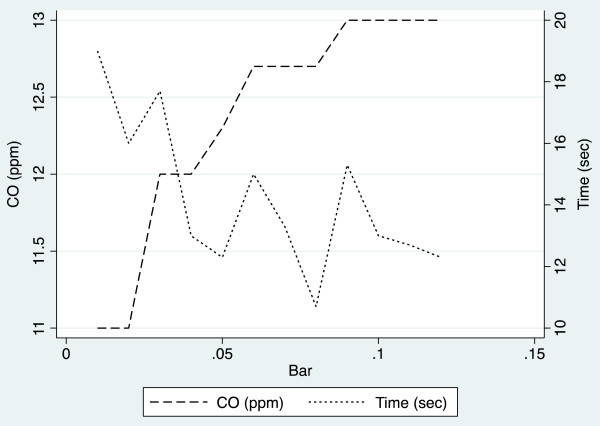
**Readings and latencies of PiCO + Smokerlyzer challenged with CO standard (9.9 ppm) at different flow rates.** Latency corresponded to the time elapsed from the beginning of expiration to the appearance of stable readings. Flows were assumed to be proportional to the pressures applied to the circuit because a linear flow could be reasonably hypothesized.

2) In vitro tests
  Table 2 reports CO values provided by B&K and PiCO+ when challenged with 4 standard CO concentrations. At 5 and 9.9 ppm, PiCO+ overestimated CO values by about 20%, while B&K maintained an excellent precision in the entire range of concentrations.
  The results obtained by challenging PiCO+ with a gaseous mixture containing 9.9 ppm of CO in nitrogen at different flow rates are reported in Figure 4. The device overestimated CO values by about 10% at low flows and by 20% or more at high flows. The time needed to achieve stable readings was 12 seconds at high flows, but increased to 19 seconds at the lowest flow tested.

**Table 2:** **In vitro CO measures (ppm) provided by B&K and PiCO+ on four standards**

Standard	B&K	PiCO+	P
**5 ppm**	5.1 (0.1)	6.7 (0.4)	<0.01
**9.9 ppm**	10.00 (0.1)	12.0 (0.9)	<0.05
**20 ppm**	19.00 (0.1)	18.5 (0.6)	0.59
**50 ppm**	47.9 (0.2)	47.0 (1.0)	0.28

Means (standard deviations) of three values are reported.

## Discussion

In this study, B&K and PiCO+ exhibited good internal consistency so that both are probably suitable for assessing the eCO variations associated with smoking status or diseases. Conversely, the eCO values obtained with the two devices were significantly different, even if they correlated very well. In particular, the B&K readings were greater than the PiCO+ at low test concentrations, and vice versa. This finding suggests that eCO values obtained with B&K and PiCO+ are not comparable and that the normal range is different.

Discrepancies between B&K and PiCO+ are hardly explained by the higher accuracy of the former because in vitro tests showed that PiCO+ overestimated CO values at low concentrations. Conversely, our data suggest that the values provided by the two devices may correspond to different phases of expiration. On this regard, B&K assessed eCO concentration 7 and 12 seconds after the beginning of expiration, i.e. in the middle and at the end of expiration, and displayed the mean of the two values. PiCO+ specifications do not report to which phase of expiration the displayed values correspond. In this study, the time needed to achieve stable readings was 12 seconds or more after the beginning of gas challenge during in vitro tests and about 15 seconds after the beginning of expiration in measurements performed on healthy volunteers. These findings suggest that the eCO concentration measured by PiCO^+^ corresponds to in the initial phase of expiration, when flow is maximum.

In most studies, eCO values have been measured with electrochemical devices, which are cheaper and more easily transportable than laser spectrophotometers, near-infrared analysers [25, 26] or photo acoustic spectrometers [22]. Among electrochemical devices, PiCO+ has been utilized both for monitoring smoking habits (which is its primary target) [27–29] and for assessing eCO levels in several diseases [8, 9, 11–17]. Normal eCO values were not exactly comparable even across studies that utilized this device, since mean values in non-smoker subjects varied from less than 1.5 [15] to 3 ppm [17]. Factors that have been proposed to explain such variability were differences in environmental CO levels, in anthropometric characteristics such as lung capacity [30] and in measurement techniques. In this regard, measures achieved during prolonged expirations provide values higher than those obtained during shorter ones [31]. High environmental CO levels can probably explain the eCO levels observed in the non smoker participants in this study (mean value 3.7 ppm) because they all lived in a big city and travelled through traffic to get to the University. A further factor that should be considered is the trend of eCO concentration during expiration, which has been divided in three phases by Schober et al. [32]. Exhaled CO concentration is zero in the initial part of the expiration (phase 1), then it increases progressively (phase 2), until a plateau is reached (phase 3). Of note, phase 2 is much less steep than in capnograms, suggesting that it may be influenced by alveolar inhomogeneities since gas from the alveoli with a longer time constant mostly contribute to the final part of expiration [33]. Interestingly, phase 3 mean eCO concentration measured by Schober et al. in a group of healthy, non-smoker volunteers, was 10.7 ppm [32], a value similar to that provided by B&K in the healthy non smoker subjects included in this study (10.9 ppm).

Differences between B&K and PiCO+ make the values obtained with one device not comparable with those obtained with the other. According to our hypothesis, the values provided by B&K may be more informative of the alveoli with a slower time constant while those obtained with PiCO+ are probably unaffected by them. Conversely, according to the latency of 15 seconds observed in healthy volunteers, the values shown by PiCO+ might even refer to phase 2 of CO expiratory trend. In this regard, alveolar inhomogeneities about CO content are probably negligible to detect smoking habit because smokers present increased eCO levels throughout the expiration. By contrast, it is not clear whether initial, mean and end tidal eCO values are equally effective as markers of respiratory or systemic diseases, so that further studies are needed. Finally, it is not clear why PiCO+ displayed eCO values lightly higher than those displayed by B&K above 30 ppm. Since the tests in vitro showed a good accuracy of both devices at those CO levels, the difference of values may hypothetically originate from a different trend of eCO concentration during deep expiration in heavy smokers. Our data suggest that PiCO+ readings can be influenced by the flow of gas through it. The more the flow, the higher the value. Hypothetically, if phase 2 is shorter in heavy smokers, readings of PiCO+ may occur at the time of maximum eCO values and maximum gas flow and this may lead to the reversal of the difference with B&K. Unfortunately, no data are available in literature in this regard.

This study has some limits. B&K and PiCO+ were only compared in healthy subjects and potential differences related to the presence of respiratory diseases were not investigated. Besides, the devices and the circuit utilized did not allow isolate measurements of eCO concentration in the last part of expiration. Finally, the devices were not compared in vivo in static conditions, for instance by collecting exhaled gas in a bag. That comparison was deemed unnecessary because the accuracy of the detectors was assessed in vitro with constant gas flows.

## Conclusions

B&K and PiCO+ can be both utilized to measure eCO because they exhibited good internal consistency and the measures obtained correlated very well. Nonetheless, values obtained with these devices are not comparable to each other and normal ranges do not coincide. Differences are probably explained because PiCO+ assesses eCO concentration in an earlier stage of expiration than B&K, which performs the analysis in the middle and at the end of expiration. Further studies are needed to evaluate which measurement technique is more effective to investigate the eCO variations induced by respiratory diseases.

## Abbreviations

eCO: Exhaled carbon monoxide
CO: Carbon monoxide
PPM: Parts per Million
HME: Heat Moisture Exchange
B&K: Brüel&Kjær 1312
PiCO+: PiCO + Smokerlyzer.
